# The Molecular Mechanisms Underlying the Systemic Effects Mediated by Parathormone in the Context of Chronic Kidney Disease

**DOI:** 10.3390/cimb46050241

**Published:** 2024-04-25

**Authors:** Minela Aida Maranduca, Cristian Tudor Cozma, Andreea Clim, Alin Constantin Pinzariu, Ionut Tudorancea, Irene Paula Popa, Cristina Iuliana Lazar, Roxana Moscalu, Nina Filip, Mihaela Moscalu, Mihai Constantin, Dragos Viorel Scripcariu, Dragomir Nicolae Serban, Ionela Lacramioara Serban

**Affiliations:** 1 Discipline of Physiology, Department of Morpho-Functional Sciences II, Grigore T. Popa University of Medicine and Pharmacy, 700115 Iasi, Romania; minela.maranduca@umfiasi.ro (M.A.M.); cozmatudor19@gmail.com (C.T.C.); clim.andreea@umfiasi.ro (A.C.); alin.pinzariu@umfiasi.ro (A.C.P.); ionut.tudorancea@umfiasi.ro (I.T.); irene-paula_popa@umfiasi.ro (I.P.P.); crislazar@ymail.com (C.I.L.); dragomir.serban@umfiasi.ro (D.N.S.); ionela.serban@umfiasi.ro (I.L.S.); 2 Division of Cell Matrix Biology & Regenerative Medicine, School of Biological Sciences, Faculty of Biology, Medicine and Health, The University of Manchester, Manchester M13 9PL, UK; roxana.moscalu@postgrad.manchester.ac.uk; 3 Discipline of Biochemistry, Department of Morpho-Functional Sciences II, Grigore T. Popa University of Medicine and Pharmacy, 700115 Iasi, Romania; nina.zamosteanu@umfiasi.ro; 4 Department of Preventive Medicine and Interdisciplinarity, Grigore T. Popa University of Medicine and Pharmacy, 700115 Iasi, Romania; 5 Internal Medicine Department, Faculty of Medicine, Grigore T. Popa University of Medicine and Pharmacy, 700115 Iasi, Romania; mihai.s.constantin@umfiasi.ro; 6 Department of Surgery, Grigore T. Popa University of Medicine and Pharmacy, 16 University Str., 700115 Iasi, Romania; dscripcariu@gmail.com

**Keywords:** chronic kidney disease, parathormone, mineral and bone disorder, diagnosis, microbiota, cardiovascular, calcification

## Abstract

Chronic kidney disease (CKD) stands as a prominent non-communicable ailment, significantly impacting life expectancy. Physiopathology stands mainly upon the triangle represented by parathormone–Vitamin D–Fibroblast Growth Factor-23. Parathormone (PTH), the key hormone in mineral homeostasis, is one of the less easily modifiable parameters in CKD; however, it stands as a significant marker for assessing the risk of complications. The updated “trade-off hypothesis” reveals that levels of PTH spike out of the normal range as early as stage G2 CKD, advancing it as a possible determinant of systemic damage. The present review aims to review the effects exhibited by PTH on several organs while linking the molecular mechanisms to the observed actions in the context of CKD. From a diagnostic perspective, PTH is the most reliable and accessible biochemical marker in CKD, but its trend bears a higher significance on a patient’s prognosis rather than the absolute value. Classically, PTH acts in a dichotomous manner on bone tissue, maintaining a balance between formation and resorption. Under the uremic conditions of advanced CKD, the altered intestinal microbiota majorly tips the balance towards bone lysis. Probiotic treatment has proven reliable in animal models, but in humans, data are limited. Regarding bone status, persistently high levels of PTH determine a reduction in mineral density and a concurrent increase in fracture risk. Pharmacological manipulation of serum PTH requires appropriate patient selection and monitoring since dangerously low levels of PTH may completely inhibit bone turnover. Moreover, the altered mineral balance extends to the cardiovascular system, promoting vascular calcifications. Lastly, the involvement of PTH in the Renin–Angiotensin–Aldosterone axis highlights the importance of opting for the appropriate pharmacological agent should hypertension develop.

## 1. Introduction

Chronic kidney disease (CKD) is a progressive and irreversible disorder defined by functional and anatomical alterations of the kidney due to a heterogeneous array of causes. The damage must be present for at least 3 months in the form of reduced filtration capacity (estimated glomerular filtration rate, eGFR less than 60 mL/1.73 m^2^/min) or biochemical damage as signaled by significant albuminuria (either over 30 mg/24 h or albumin-to-creatinine ratio (ACR) higher than 30 mg/g) or hematuria. CKD prevalence, despite high figures, is likely underestimated due to the silent clinical picture in the early stages [1]. In fact, approximately 90% of subjects eligible for CKD diagnosis are blissfully unaware of their potential condition, and only 50% of the patients with drastically low eGFR have acknowledged their diagnosis [2,3]. The very high and increasing trend in the prevalence of kidney disease is augmented by improved patient survival, better screening and diagnosis protocols (employing markers such as creatinine, cystatin C, albuminuria), along with the wide variety of risk factors, among which diabetes and hypertension account for two-thirds of all cases [4,5,6]. 

The comorbidities brought along with the kidney disease are more relevant to the patient’s prognosis since only a small fraction of them eventually develop end-stage renal disease (ESRD) [7]. This applies mainly to subjects with mild to moderate reduction in eGFR. However, cardiovascular events are the most prevalent cause of morbidity in CKD patients across all stages of the disease [8]. Traditional risk factors for cardiovascular disease (CVD)—dyslipidemia or hypertension—are unable to account for the high rate of mortality in CKD subjects. Therefore, the non-traditional risk factors must complete the cardiovascular picture. An altered mineral metabolism, a low-grade persistent state of inflammation, altered microbiota, oxidative damage and the invariable presence of endothelial dysfunction are all characteristic of ongoing chronic kidney disease [9].

Renal function decline is rapidly followed by a tendency towards inorganic phosphate (Pi) retention. This tendency is counterbalanced by the intervention of the axis parathormone (PTH)–Vitamin D–Fibroblast Growth Factor-23 (FGF-23). Alteration in the activities of each component should prevent Pi retention, meaning that any overshot out of the normal Pi range signals the definite installation of the disease. 

The current review aims to highlight the particular mechanisms employed by parathormone in the context of the PTH–Vitamin D–FGF-23 axis in determining multiple organ damage characteristics in CKD patients. PTH exhibits higher than normal levels in CKD as early as the G2 stage [10]. While embryological development and homeostasis of multiple organs—small and medium-diameter blood vessels, cardiomyocytes, and bone tissue—is dependent on the presence of PTH, persistent exceedance of normal concentrations leads to rapid tachyphylaxis and reversal of any beneficial effect. One of the therapeutic objectives, especially in the higher stages of CKD, is lowering the levels of PTH while maintaining slightly above the normal range. Paradoxically, however, the skeletal tissue might benefit from pulse therapy even under persistently high PTH levels.

## 2. Materials and Methods

For the assembly of this narrative review, we performed a computer literature search on PubMed/MEDLINE/PMC, Embase and Wiley databases, including terms for PTH, parathormone in association with chronic kidney disease (CKD), and any of the following: mineral and bone disorder (MBD), diagnosis, microbiota, cardiovascular, and calcification. All relevant articles published between 1 January 2004 and 30 March 2024 were reviewed in detail, and duplicates were removed. The recovered entries were limited to data involving human patients with demonstrated and persistent hyperparathyroidism, as well as animal models of chronic kidney disease. Breakthrough articles published before 2004 were also included. The identified data were then thoroughly screened independently by two authors, by title and abstract. Only English-written manuscripts were included. A narrative review was performed, structured upon linking the clinical and paraclinical observations in human patients to the molecular mechanisms underlying the effects of PTH.

## 3. Parathyroid Hormone Physiological Actions

Parathormone is the secretion product of the parathyroid glands and is mainly involved in regulating calcium (Ca) and phosphate concentrations in the extracellular milieu. PTH and its paracrine effector PTH-related protein (PTHrP) interact on target tissue with the G-protein PTH receptor type 1 (PTHR1). The most powerful regulator for PTH release is serum ionic calcium, which, upon binding to the calcium-sensing receptor (CaSR), inhibits the release of PTH. Conversely, a drop in Ca leads to stimulation of parathyroid glands to synthesize and release PTH. Restoration of calcium balance occurs by inhibiting phosphate and bicarbonate renal reabsorption, together with increased reabsorption of bivalent ions (calcium and magnesium) [11].

In the classical view, parathormone determines its effects on bones, kidneys and small intestine. In bones, PTH signaling is the prototype for osteoclastogenesis [12]. Canonical activation of PTHR1 in osteoblasts leads to upregulation in the expression of receptor activator for nuclear factor kappa-B ligand (RANKL), essential in differentiation to osteocytes. Osteoprotegerin, a powerful inhibitor of RANKL and a barrier against bone dissolution is negatively regulated by PTH. The newly formed osteoclasts would remodel the skeleton by degrading hydroxyapatite, releasing calcium into the bloodstream and accomplishing parathormone’s fundamental effect. Serum calcium is indirectly enhanced by an increase in Vitamin D (calcitriol, 1,25-dihydroxycholecalciferol) activity. PTH accelerates synthesis of the enzyme involved in the rate-limiting step in calcitriol synthesis, 1α-hydroxylase. The resulting increase in Vitamin D axis activity leads to better absorption of calcium in both proximal and distal intestinal segments [13].

PTH acts on renal mineral handling at three distinct levels. Calcium reabsorption is enhanced by PTH in the distal convolute tubule and collecting duct through the regulation of a highly selective calcium channel TRPV5 (transient receptor potential cation channels). The expression of TRPV5 is also dependent on extracellular calcium concentration through a calcium-sensing receptor located on the luminal side of the tubule, which closely resembles the one found in the parathyroid gland [14]. An increase in circulating Ca could pose a threat to the precipitation of calcium phosphate; therefore, PTH decreases the reabsorption of phosphate at the proximal convoluted tubule, allowing for higher ionic calcium concentrations. Phosphate reabsorption occurs mainly across the membrane of the proximal convoluted tubule through sodium-dependent cotransporters NaPi-IIa and NaPi-IIc. Close proximity and tight enzymatic interaction between PTHR1 and NaPi-IIa are ensured by sodium–hydrogen exchanger regulatory factor-1 (NHERF1). PTH binding leads to phosphorylation of NHERF1, lowering the membrane stability of the sodium-phosphate cotransporter and ultimately downregulating Pi reabsorption in the proximal tubule [15].

## 4. Chronic Kidney Disease Rapidly Leads to Mineral and Bone Disorder

Aside from being one of the most common chronic diseases, CKD is currently one of the leading non-communicable causes of death. The estimated 843 million patients in all stages of CKD in 2019 does not reveal the whole extent of the phenomenon [16]. There is a higher record of patients in stages 3–5 compared to earlier phases, with the latter accounting for less than 75% of the cases, likely due to the clinically silent nature of CKD in its incipient phases. The high prevalence of CKD is further grieved by an unforeseen increase in the relative death rate compared to 1990 [17]. Apart from the characteristic drop in the glomerular function (from stage 2 onwards), cardiovascular (CV) complications play a major role in the extent of all-age mortality driven by CKD. Population-based studies have drawn attention to this specific issue after demonstrating an increase in CV mortality in patients presented with either GFR lower than 60 mL/min/1.73 m^2^ or albuminuria—both indicators of advanced-grade CKD [18]. 

Mineral bone disorder, also known as CKD-MBD in the literature, envelopes the changes in biochemical and hormonal profiles responsible for increased CV events and mortality, faster CKD progression rate, and susceptibility to fractures. The three main pillars founding MBD are as follows: (a) altered biochemistry with respect, but not limited to, calcium, phosphorus, PTH and Vitamin D; (b) abnormal bone turnover rate, linear growth, or lower sturdiness; (c) vascular or another ectopic site calcification [19]. Phosphate is the central element in the development of MBD and is mainly regulated by kidneys, parathyroid glands and intestine [20]. Early in the course of CKD, arising from the reduced number of functional nephrons, the remaining undergo adaptative changes in response to the overall reduced filtration capacity. 

### 4.1. Classical View of CKD-MBD

Classically, the progressive loss of renal function required adaptation of the remaining ones, otherwise stated as the “intact nephron hypothesis” [21]. The adaptive processes consist of increasing the single nephron filtration rate, lowering the reabsorption rate, and simultaneously increasing tubular secretion. These changes would create the premises for the uremic state, the “trade-off” of renal function decline [22]. Prior to the unravel of FGF-23, phosphate retention was considered the main culprit for secondary hyperparathyroidism in patients with renal function decline. The increase in serum Pi drives a reduction in the final hydroxylation step in the synthesis of 1,25-dihydroxyvitamin-D3 (calcitriol, part of the wider Vitamin D group of substances). The drop in active Vitamin D, together with hyperphosphatemia, leads to hypocalcemia. The disturbed Ca/P balance, along with the reduced activity of Vitamin D, represent powerful stimuli for PTH secretion, which in turn upregulates phosphate excretion, at least until the very end stages of renal dysfunction. Some authors noted a lower activity of the Vitamin D axis in the absence of Ca/P imbalance, suggesting that calcitriol is a more powerful stimulus and could be the trigger for the increased PTH secretion [23]. The “trade-off hypothesis”—that is, secondary hyperparathyroidism (SHPT) develops in response to the tendency of phosphate retention—is strengthened by the fact that in some cases Pi level is normal until advanced CKD stages [24]. 

Homeostasis is maintained with regard to phosphorus until the highest stages of CKD at the expense of alterations in levels of PTH and FGF-23, with calcitriol enclosing an interdependent hormonal triangle. Lastly, FGF-23 coreceptor Klotho (Kl) plays a role in molecular mechanisms commonly encountered in advanced-age phenotype, reinforcing the consideration of CKD as a progeric disease. The emergence of these newer hormones allowed for an updated perspective of the classical “trade-off hypothesis” view of CKD.

### 4.2. Updated “Trade-Off Hypothesis”

#### 4.2.1. FGF-23, the Missing Piece from the Puzzle

The discovery of FGF-23 has brought massive benefits to understanding the development of SHPT. Firstly isolated in the ventrolateral thalamic nuclei of rodents, FGF-23 is the newest member of the endocrine subfamily of fibroblast growth factors [25]. The main stimuli for FGF-23 synthesis are oral phosphate loading, increased circulating levels of 1,25-dihydroxyvitamin-D3 and PTH. Reduced ionized calcium, depletion of iron deposits, and oxidative and inflammatory stress are additional regulators of FGF-23 production and secretion [26]. Canonical signaling of FGF-23 is achieved through FGF-23 receptors (FGFRs) in the presence of the Klotho coreceptor molecule [27]. Non-canonical signaling takes place in the absence of Kl but requires higher levels of FGF-23 due to the lower affinity to receptors.

In humans, FGF-23 increases renal phosphate excretion on the proximal tubule by lowering the expression of luminal sodium-dependent Pi cotransporters, resulting in a lower reabsorption rate [28]. FGF-23 secretion is more sensible than PTH to Pi retention since the former hormone exhibits levels out of the normal range earlier than PTH over the course of the disease [29]. The relationship between FGF-23 and Vitamin D is dual-way since not only does calcitriol upregulate FGF-23 secretion, but also FGF-23 suppresses the final hydroxylation step of calcitriol synthesis and promotes its degradation through 24-hydroxylase [30]. This inhibitory effect translates to the gastrointestinal system, where FGF-23 indirectly reduces calcium and phosphate absorption through the inhibited Vitamin D axis activity. On an intestinal level, FGF-23 directly downregulates the expression of luminal sodium-dependent Pi cotransporter NaPi2b, similar to the proximal renal tubule [31].

#### 4.2.2. Uremic Conditions Diminish the Potential Benefit of Increased Circulating FGF-23

**Figure 1:**
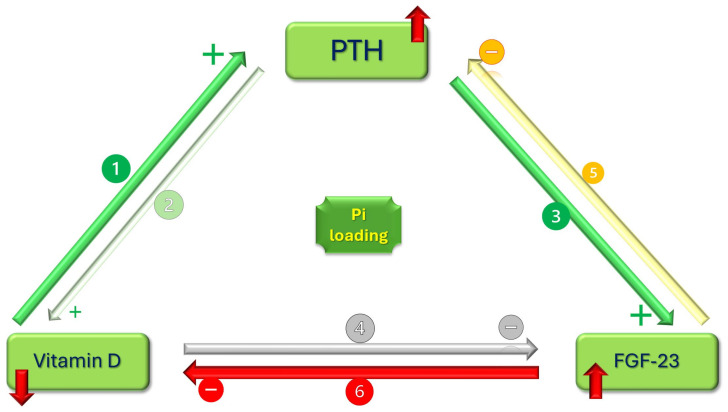
Summary of the hormonal alterations defining CKD-MBD. PTH, parathormone. FGF-23, Fibroblast Growth Factor-23; Pi, plasma phosphate. Overall, there is lower activity of Vitamin D but higher plasma levels of both PTH and FGF-23. The drop in Vitamin D synthesis in response to the trend in Pi drives PTH secretion (process 1), which then directly influences Vitamin D synthesis and FGF-23 secretion (arrows marked 2 and 3, respectively). The drop in Vitamin D is not a powerful inhibitor of FGF-23 secretion (process 4). However, the elevated FGF-23 drastically reduces Vitamin D hydroxylation (process 5). Lastly, under uremic conditions, FGF-23 does not efficiently reduce PTH release (arrow numbered 6).

FGF-23 exhibits a different effect on parathyroid glands in CKD patients compared to healthy subjects [32]. In normal rodent specimens, the addition of FGF-23 under low calcium conditions led to a lower and blunted increase in PTH secretion and inhibition of parathyroid cell hyperplasia. Immunohistochemical evaluation of these normal cells showed an increase in both Vitamin D receptors and calcium-sensing receptors, which are responsible for the regulation of PTH synthesis. The expression of the two former receptors paralleled the activity of the extracellular signal–regulated kinase 1/2 (ERK1/2)–mitogen-activated protein kinase (MAPK) pathway. FGF-23 has been previously shown to increase the activity of the ERK/MAPK axis through canonical signaling [33]. The ameliorated increase in PTH secretion in conditions of hypocalcemia can, therefore, be attributed to the artificial introduction of FGF-23 in the milieu of normal cells. However, neither of these effects was reproduced in parathyroid cells originating from uremic rats, and there was not a significant difference regarding in vivo versus in vitro conditions. A decrease in FGFR expression provides an explanation for the observed lack of effect in the cells of uremic rats. Uremic toxins, with the exception of phosphate, could be responsible for a lower expression of FGFRs and/or Kl. Parathyroid gland cells originating from healthy rodents were placed in a high phosphate milieu, but the FGF-23 was still efficient in reducing PTH expression, ruling out phosphate as the critical uremic toxin. Persistent exposure to either hypocalcemia or high FGF-23, conditions which are often simultaneously met in advanced CKD patients, leads to lower expression of FGFR and Kl. Lastly, the higher turnover rate in SHPT could lead to a lower expression of FGFRs per cell. These arguments likely extend to human patients, pointing towards the trend exhibited by FGF-23 and PTH in the earlier stages of CKD. Lower-stage patients tend to benefit from the inhibition potential of FGF-23 on PTH, therefore accounting for the initial increase in FGF-23, followed later similarly by PTH [29]. A summary of the noted hormonal changes characterizing CKD-MBD is depicted in Figure 1.

#### 4.2.3. Klotho—An Aging-Related Molecule

One of the features occurring early in the progression of CKD is an imbalance in the Klotho/FGF-23 axis [2]. A lower circulating level of Kl and higher FGF-23 lead to imbalanced signaling, favoring the expression of non-canonical effects. The phosphaturic canonical outcome on the renal proximal tubule is greatly reduced in conditions of Kl depletion, as demonstrated in dialysis patients [34]. These patients also demonstrated both very high PTH and FGF-23 levels. Relative Klotho depletion, as is the case in CKD individuals, yields a lower affinity of FGFRs towards FGF-23. Together with an already reduced potential of FGF-23 in parathyroid gland cells in uremic conditions, the reduced specificity of FGFRs negatively contributes to an even lower potential of FGF-23 to reduce PTH secretion [32]. Considering the progeric and uremic conditions characterizing progressive kidney disease, once the PTH levels have taken off the normal range, there are presumably few physiological mechanisms left in order to attenuate the inevitable development of secondary hyperparathyroidism [35]. 

#### 4.2.4. Vitamin D Encloses the Hormonal Triangle

Calcitriol is the active form of Vitamin D synthesized in the human body, and its balance depends on two enzymes: 1-α-hydroxylase, mainly restricted to the kidney, involved in the last and rate-determining step of the synthesis, and 24-hydroxylase related to its inactivation [36]. Calcitriol binds the Vitamin D receptor in the cells of target organs, leading to a conformational change and preferential coupling with the retinoid X receptor. The new complex translocates into the nucleus, where it alters the expression of certain genes pertaining to its effects [37]. 

Synthesis of 1-α-hydroxylase—D3 (or cholecalciferol) increase—is enhanced by PTH and hypocalcemia, while hyperphosphatemia and FGF-23 accelerate D3 degradation [30]. Under normal circumstances, calcitriol acts on the parathyroid gland cells directly by inhibiting PTH synthesis and on the intestine by increasing the absorption of both calcium and phosphorus. Serum phosphate increase should, therefore, upregulate PTH, but the increase in ionic calcium is a more powerful stimulus on the parathyroid gland, resulting in a decrease in PTH synthesis in line with the direct effect on the gland cells [36]. Calcitriol acts on bone tissue by increasing the synthesis of FGF-23, closing a feedback triangle of the three hormones (Figure 1). 

Since phosphate homeostasis is generally maintained until the more advanced stages of CKD, the main determinants of calcitriol level are PTH, FGF-23 and functional renal mass. 1-α-hydroxylase, the rate-limiting enzyme pool, is sufficient until end-stage renal disease, claiming PTH and FGF-23 as the principal determinants of calcitriol levels. Cross-sectional CKD population studies have determined an inverse relationship between PTH and calcitriol levels, albeit redeeming FGF-23 as the more significant ground for Vitamin D axis activity [38]. Furthermore, due to the higher impact bared by FGF-23 on calcitriol and the earlier stage at which this hormone surpasses the normal range, compared to PTH, the alterations in Vitamin D activity occur earlier than the ones in PTH circulating level [39]. 

## 5. Biochemical Diagnosis of CKD-MBD: Focus on PTH

KDIGO guidelines recommend beginning the monitoring of CKD patients starting from stage G3a in adults and G2 in children. Recommended parameters include calcium, phosphate, PTH and alkaline phosphatase, which is a reliable marker for bone tissue turnover [19]. Since PTH is among the last hormones to increase in response to renal function decline, an abnormal PTH level indicates an already advanced stage of CKD. 

FGF-23 was proven to be the first hormone to respond in the “updated trade-off hypothesis” and could serve as an earlier marker for kidney disease. The biological variation in FGF-23 levels, the sum of within-subject and between-subject variations, was proven small enough to allow for a reference range to be established, including both healthy and dialyzed populations [40,41]. However, the majority of existing determination kits are deemed ‘research only’, rendering them useful only for in-vitro studies [42]. In addition, the existing kits do not separately quantify the intact and fragments of FGF-23, further reducing the clinical relevance of this parameter. Neither does assessment of calcitriol prove suitable for CKD progression and risk of developing serious complications since Vitamin D levels are heavily influenced by nutrition [43]. 

Addressing the issue encountered in FGF-23 measurements—the inability to distinguish between intact hormone or fragments—there was a serial development of radioimmunoassay (RIA) kits. First-generation RIA was based on the recognition of the mid portion of the PTH molecule, leading to overestimated levels due to degradation fragments [44]. The second-generation RIA utilized an updated antibody designed to recognize the full length of the PTH chain, especially the (1–6) fragment. While the third and latest RIA version displayed higher PTH specificity and more accurate results, the second-generation RIA is the most widely used today [45,46]. Neither third-generation assay alone nor combined use of the previous ones displayed a statistical improvement in diagnostic value, advancing the use of a single determination of PTH by second-generation RIA as clinically significant and sufficient [47,48].

Baseline PTH measurements, a single value at the beginning of a follow-up study, returned great statistical results with respect to the complication rate. CKD patients who had undergone parathyroidectomy displayed a lower incidence of fractures compared to the matched control group [49]. As such, the surgical approach of SHPT is a method for the initial drastic lowering of PTH levels. Even more noteworthy is the association between baseline PTH levels and cardiovascular complications among populations without apparent renal disease [50]. However, practitioners should not conclude the severity of MBD based on a single measurement. The frequency of monitoring the biochemical parameters and PTH levels should be based on the magnitude of detected abnormalities and the progression of renal function decline [19]. The diagnostic and management approach of MBD is based on the trend in changes in the relevant markers, with special emphasis on the PTH level [51]. There is a statistically significant relationship between the trend in multiple MBD markers and defined renal endpoints (>30% reduction in GFR, necessity for dialysis or transplantation). PTH, serum calcium and phosphate are independently correlated with progression to kidney failure in CKD patients staging from as early as G2 up to G5 end-stage disease [10].

Despite FGF-23 being an earlier modifiable marker over the course of CKD, PTH level still remains the most used assay in these patients. Practicians should record the value of plasma PTH at the first presentation of the patient, along with serum calcium and phosphate. Observing how far outside the normal range these parameters are could provide a projected rate of renal disease progression, guide the extent of needed therapeutical intervention and increase the frequency of monitoring the patient’s laboratory analysis.

## 6. Intestinal Microbiota in Mediating PTH Actions on Bone Turnover

### 6.1. Intestinal Microbiota Acts as Vehicle in Mediating Bone Loss in Hyperparathyroidism

Emerging evidence on rodent models highlights the role played by the microbiota in establishing the effect played by PTH on bone tissue—resorption or formation [52,53,54]. Helper T cells subtype 17 (Th17) presence in bone marrow is required and sufficient in mediating the osteolytic effect of PTH [54]. Short-stranded filamentous bacteria (SFB) are necessary and sufficient to enable PTH to induce the production of TNF from T cells. Local collaboration of SFB and the previously activated TNF-producing T cells initially expand the TH17 population on a local scale. Intestinal TNF-producing cells then migrate to bone marrow, where they are the ideal homing environment for Th17 through the expression of CXCR3 (a Gαi protein-coupled receptor, member of the CXC chemokine receptor family) and CCL20 (a strongly chemotactic cytokine for lymphocytes). Egression and migration of intestinally produced Th17 into bone marrow are essential in accomplishing the resorptive action of PTH since TNF-positive T cells alone yielded no effect. The absence of SFB through antibiotic treatment yielded no effect of exogenous PTH. Strategies for preventing excessive colonization of SFB responsible for the origin of Th17 or modulating Th17 cell trafficking remain, however, in future research subjects.

### 6.2. Short-Chain Fatty Acids Are Fundamental in Mediating PTH-Dependent Bone Formation

In healthy humans, a significant proportion of the microbiota is represented by short-chain fatty acid (SCFA), producing bacteria, the central stone in the complex diet–gut–brain axis. These genera produce three valuable metabolites: acetate, propionate and butyrate. The latter has demonstrated great benefit in CKD due to its potential to reduce insulin resistance in diabetic kidney disease, ameliorate inflammation and oxidative stress, and improve cardiovascular protective capacity [55,56,57]. 

Circulating butyrate is also key in mediating the effects of PTH on the skeleton, such as bone formation [52]. Intermittent administration of PTH mimics the physiological secretion and should induce the known and beneficial osteogenic effect. Treatment with oral antibiotics led to a 50% reduction in circulating butyrate and hindered any action of PTH, and neither bone formation nor resorption was noted in female rodents [52]. Single-dose supplementation with butyrate on the same antibiotic-treated animals restored the function of PTH, highlighting the importance of this bacterial metabolite alone. Healthy gut flora, both in mice and humans, induces differentiation of CD4+ T cells into regulatory T cells (Tregs) both through direct stimulation by microbiota and diet-derived factors and co-stimulation by dendritic cells activated by similar antigens [58,59]. SCFAs derived from undigestible carbohydrates similarly promote differentiation into Tregs locally, but essentially also in other human organs [60]. Butyrate targets T cells and dendritic cells since the deletion of PTH receptors on T cells does not blunt the anabolic response to intermittent PTH [61]. The resulting higher population of Tregs stimulates CD8+ T cells located in the bone marrow to upregulate the gene for Wnt10b. This works as an agonist ligand in the Wnt (Wingless and Integrin-1 portmanteau) osteogenic pathway, occupying the orthosteric site and preventing the binding of inhibitors such as sclerostin [62]. 

The overall anabolic effect of intermittently administrated PTH is predominantly determined by the direct stimulation of PTH receptors on the surface of osteocytes and, to a lower extent, by competitive inhibition of the Wnt-blocking ligands. However, at the very least in mice, both pathways are required to be activated simultaneously in the event of PTH stimulation since blocking the PTH receptors on osteocytes hinders the activity of PTH to the same extent as blocking the butyrate/Wnt10b/Wnt pathway [63].

### 6.3. Damaged Gut Microbiota Takes a Toll on Bone Homeostasis: Can Probiotics Save It?

The uremic state, typical of advanced-stage CKD patients, impacts gut health. The pro-inflammatory and pro-oxidative conditions in the gastrointestinal system start from the duodenal segment, generating a disruptive cycle against healthy bacteria [64]. Recent studies provide insight into a strong correlation between lower SCFA concentrations and reduced SCFA-producing bacteria [65,66]. Regarding butyrate specifically, results are yet conflicting regarding the association of more advanced CKD to a lower butyrate serum level [66,67,68]. Despite the advances in understanding nutrition needs in CKD, patients may experience difficulty in following recommendations due to slower gastric emptying and reduced appetite, especially in the later stages of the disease. This reduces the theoretical benefit of a recommended diet.

The impact of beneficial bacterial metabolites is apparent in the results yielded by certain probiotics. During an experimental study on healthy rodent subjects, oral delivery of Lactobacillus rhamnosus GG (LGG) alone led to a 2-fold increase in trabecular bone mass through a pathway seemingly very similar to butyrate [69]. Since exogenous administration of PTH yields no effects in the absence of physiological levels of butyrate, the latter exerts a more powerful impact on the overall health status of the bone than PTH does. Other species of Lactobacillus proved beneficial under the pro-inflammatory intestinal conditions due to the uremic milieu in advanced stages of CKD, stating that the production of SCFAs as central in reducing the injury- and fibrosis-related proteins [70,71,72]. Unfortunately, these results are based only upon rodent model research, with very limited proof on human subjects. With the exception of butyrate, caution is advised when interpreting the impact borne by SCFAs in relation to PTH-mediated osteogenesis since SCFAs generally exhibit only a T-cell-independent antiresorptive function without influencing the formation of new bone mass [73].

**Figure 2:**
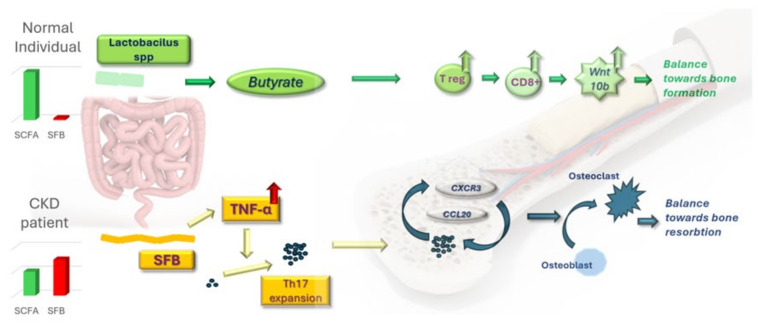
Intestinal microbiota influences the course of PTH action. SCFA, short-chain fatty acid-producing bacteria; SFB, short-strand filamentous bacteria; TNF-α, Tumor necrosis factor-α; Th17, subtype 17 of helper T cells; T reg, regulatory T cells; CXCR3 and CCL20, homing molecules for TH17 inside of bone marrow; CD8+, subtype CD8+ T cells; Wnt10b, agonist ligand of Wnt pathway on bone tissue. Green upwards arrows denote upregulation in beneficial steps towards bone formation. Red upwards arrow denotes increase in catabolism-priming factor TNF-α.

Restoring beneficial gut flora in the absence of exogenous PTH intake proved promising results on bone formation. CKD is fundamentally characterized by elevated PTH, starting from as early as stage G2 onward. External pulse-administration of PTH mimics the intermittent PTH profile in an attempt to rescue the anabolic bone status and to reduce hyperphosphatemia [74,75,76]. By ensuring physiological levels of circulating SCFAs, healthy intestinal microbiota would potentiate the benefit of intermittent PTH, but current data are sparse. A simplified summary of the main mechanisms occurring on an intestinal level is depicted in Figure 2.

The profile of a CKD patient is characterized by inflammation and uremic toxicity. Together, these lead to an imbalanced intestinal microbiota. The prevalence of short-stranded filamentous bacteria is enough to kickstart an osseous resorptive cascade. Conversely, the preponderance of short-chain fatty acid-producing bacteria inhibits and even reverses the dissolution process independently of PTH levels. While a fiber-rich diet could help the beneficial bacterial strands thrive, probiotic administration, including Lactobacillus rhamnosus GG, may be necessary to rescue the healthy flora.

## 7. Secondary Hyperparathyroidism and Bone Homeostasis

### 7.1. PTH Signaling Exhibits a Dual Behaviour in Bone Tissue

The ultimate role of parathormone is the re-establishment of normal serum calcium concentrations, amongst other mechanisms, by dissolving hydroxyapatite from skeletal structure. PTH displays a dichotomous action on bone homeostasis dependent on the infusion regime: intermittent infusion leads to anabolic effects, while continuous administration leads to bone loss. The classical coupling of intact PTH (1–34) to PTHR1 yields downstream activation of protein kinases A and C pathways (PKA and PKC, respectively). Terminally truncated PTH analogs shed light upon the influence played by each kinase in response to continuous and intermittent administration. Separate in vivo activation of either of the axes does not lead to catabolic effects to the same extent as continuous administration of the intact hormone; this suggests a downstream interaction between PKA and PKC in determining the catabolic potential. Conversely, an artificial intermittent increase in PKA activity displayed matching anabolic effects to intact PTH, advancing PTH (1–31) as a possibly more efficient treatment option with a wider therapeutical safety range [77].

PTH binding to its receptor on the surface of osteoblasts determines a shift in gene expression toward transition to osteocytes. One of the crucial molecules in this process is RANKL, upregulated in vivo by the PKA pathway. Despite both intermittent and continuous infusion of PTH leading to an increase in RANKL, the discontinuous regime yields transitory and progressively much higher levels. In order to achieve the anabolic effect, a preceding transient activation of osteoclastic activity is required in order to prime the subsequent deposition sites [77]. Increased PKA activity yields an increased cAMP response element modulator (CREM), which in turn exhibits a negative regulatory function towards osteoclastogenesis in intermittent PTH injections [78]. Phosphorylation of Runx2 occurs in a similar PKA-dependent manner downstream of PTHR1 activation, enhancing transcription of the anti-apoptotic gene Bcl-2 and enabling prolonged anabolic signaling [79]. In order to balance the osteogenic effect, PTH limits osteoblast survival by enhancing the activity of the Smad ubiquitin regulatory factor, part of proteasome targeting Runx2.

### 7.2. Recent Advances in PTH Signaling Mechanisms with Respect to Bone

In response to renal injury, several pre-programed nephrogenic mechanisms, such as the Wnt pathway, are reactivated in order to maintain the remaining cells in a constantly activated state. The downstream signaling cascade and outcome on renal homeostasis are heavily influenced by the binding ligand, awarding the Wnt pathway a dichotomous outcome as exhibited by PTH/PTHR1. Individual coupling of members from the Dickkopfs family (Dkk) yields a favorable prognosis following injury. However, systemic pathological activation of the Wnt pathway, despite promoting regeneration, inhibits definitive differentiation and acquirement of full cellular capacity, overshadowing the prognosis of CKD [80].

#### 7.2.1. Dkk1 as an Intermediate Marker of PTH Signaling

Nephron damage (early in the development of CKD) induces upregulation of the Wnt pathway. In turn, primed Wnt determines in canonical fashion increased Dkk1 transcription, which acts in a negative feedback manner on pan-Wnt activity [81]. Dkk1 levels increase early in the course of the disease, mainly under the stimulus of tubular epithelial proliferation. With advancing disease, as measured through eGFR, Dkk1 circulating levels progressively decline but never return to the normal range [82,83]. More importantly, the extent of Dkk1 concentration decline is independently and inversely associated with PTH levels in CKD subjects. PTH normally requires intact Wnt signaling in order to exhibit its full actions, but inhibition of Wnt by overexpressed Dkk1 did not completely impair PTH actions [84].

Circulating Dkk1 levels are independently and inversely associated with bone mineral density. This holds true in comparative populations of osteoporotic patients—yet otherwise healthy—versus control subjects, an association that remained significant even after age was computed [85]. Type 1 diabetic profile induces a profound increase in circulating Dkk1. The role of Dkk1 in the pathogenesis of CKD-MBD should not be underestimated since the association of a Dkk1-antibody with a phosphate binder prevents and reverses the vascular calcification in early stages of CKD and normalizes FGF-23 levels, therefore completing the picture of CKD-MBD [82].

#### 7.2.2. Sclerostin Regulation—Downstream Cascades in PTH Signaling May Be More Powerful Than PTH Itself

Sclerostin is another inhibitor of the Wnt pathway, a powerful antagonist of Wnt and bone morphogenetic proteins, more selective than Dkk1 [86]. Alongside Dkk1, sclerostin displays increased circulating levels in CKD patients, although there is vast variance across the analyzed groups [83,87,88,89,90]. Contrasting with Dkk1, sclerostin shows an increasing trend with a decline of eGFR, independent of PTH levels [83]. However, assessment of the effect bared by PTH alone on sclerostin returned dissimilar results to the ones observed in CKD. Continuous PTH expression on osteocytes resulted in reduced expression of the Sost gene, responsible for the end-product sclerostin [62,84,91]. Despite sclerostin being a useful marker in bone disease, the lack of correlation with PTH levels and the apparently opposite effect in CKD conditions compared to an isolated alteration in PTH concentration suggest the possibility of an intermediate regulatory level. Increased activity of Dkk1 is paralleled by a corresponding increase in sclerostin serum levels, while neutralization of the former abolishes the concentration of sclerostin [82,92].

#### 7.2.3. PTH Is the Central Element in Two Progressively Aggravating Entities with Systemic Reverb: Renal Osteodystrophy and Cardiovascular Disease

Bone strength is determined by density, quality, and turnover. The activity of bone remodeling units (osteoblast/osteoclast coupling) is responsible for maintaining the great resiliency of the bone in adult life. However, a decline in bone renewal ability starts between the fourth and fifth decade of life. Adding to classical risk factors for fracture—age, gender, low body mass index, current smoking and fragility in first-degree relative—the alterations in mineral balance and bone homeostasis contribute to CKD patients [93]. The abnormally high level of PTH early in the course of CKD projects two progressive outcomes: renal osteodystrophy and cardiovascular disease. Renal osteodystrophy includes three main entities: osteitis fibrosa cystica, osteomalacia and adynamic bone disease [94]. Despite presumably preventing osteoclastogenesis, PTH over-suppression renders a lack of signaling in osteoblasts, resulting in drastically low bone turnover—adynamic bone disease. This condition has been statistically increasing in recent years due to the widespread and clinically indiscriminate use of Vitamin D, calcimimetics, and even phosphate binders [95]. Conversely, consistently high levels of PTH may lead to the development of osteitis fibrosa, characterized by persistent osteoclastic activity and bone resorption, converging to a tremendous risk of fractures [96]. Prolonged resorption can induce the formation of regions with severe bone loss, fibrous replacing of which results in the so-called “brown-tumor”. These radiographic lytic lesions should not be mistaken for metastasis. Osteomalacia is possibly the least severe of the three, arising from adequate use of medication aiming at reducing PTH levels. The reduction in PTHR1 signaling is expected to reduce (but not abolish) bone turnover and mineralization but, conversely, increase fracture risk [94].

#### 7.2.4. Fracture Risk Is an Indirect Measure of Cortical Bone Mass and Mineralization Efficiency

The main determinants in the assessment of the risk of fracture in humans are bone mineral density, bone turnover and biochemical markers for bone formation and resorption. Fracture risk is moderately correlated with PTH serum concentrations but not in the expected linear fashion. PTH concentrations more than three times the upper range limit showed significant association with the risk of new fracture, but so did CKD patients exhibiting PTH levels in the normal range, albeit significantly lower than expected for a subject with end-stage renal disease undergoing dialysis [97,98]. These conclusions were strengthened by later studies on cohorts of various CKD stages or hemodialyzed patients, where a significant risk for fracture development was determined by extreme values of PTH, outside of the expected range in an advanced renal disease subject, but not necessarily outside of the range of a healthy individual. There is plenty of recent clinical evidence promoting the utility of serum PTH in assessing non-adequate bone turnover, with most studies advancing two cut-off values for differentiating low versus non-low and high versus non-high turnover. Furthermore, PTH exhibited good or modest predictability of bone homeostasis against either classical markers of bone turnover (bone alkaline phosphatase) or newer and more expensive markers such as Activin and Tartrate-resistant acid phosphatase (TRAP) 5b [99,100,101,102,103,104,105].

Kidney transplantation should break the vicious cycle of CKD-MBD biochemical derangements. Indeed, a consistent drop in PTH circulating levels is reported up to 6 months following transplantation [106]. However, in spite of the decreasing trend, PTH levels remain out of the normal range, particularly during the first year after intervention in 60% of patients [107,108]. One of the mechanisms underlying the increased risk of fractures even after transplantation is persistent hyperparathyroidism (HPT), which in turn could be the sum of SHPT and de novo HPT. SHPT development is influenced by the duration of dialysis, the duration of HPT prior to transplantation, and the presence of nodular hyperplasia of the parathyroid gland [109]. De novo HPT is a different face of the deteriorating renal graft under a load of altered mineral homeostasis and several MBD markers. Histological preparations of bone specimens from patients who have undergone transplantation, in some cases, reveal a stall of bone deposition only 6 months after surgery. This may be interpreted as adynamic bone disease and was significantly correlated with abnormally low PTH levels, again highlighting the biphasic course of action of this hormone [106]. In addition, the double-edged sword symbolizing PTH concentration with respect to bone turnover advances measurements of this hormone as being essential in predicting the evolution of CKD-MBD. Lab quantification of PTH becomes especially relevant starting from stage 3 CKD since, from this point onwards, in the course of the disease, PTH is most likely increased in response to the trend in phosphate loading [110].

PTH displays a dual behavior upon bone tissue mass, depending on the concentration profile. The course of PTH action is decided by the predominant Wnt ligand, the two most representative being Dkk1 and sclerostin, although pharmacological intervention at this level is not currently standard. Persistent levels of PTH, as in CKD-MBD, lead to the development of renal osteodystrophy. As tempting as a drastic reduction in serum PTH may be, this could completely abolish mineral turnover and osteoblastic cell activity. Current recommendations advise maintaining serum PTH slightly higher than normal. The more controlled the PTH serum is, the lower the fracture risk in the CKD patient.

## 8. Parathormone Role in Determining Cardiovascular Disease: Friend or Foe?

### 8.1. Physiological Benefits of PTHR1 Activation—Antiapoptotic Behaviour and Systemic Vasodilatory Potential

PTH/PTHrP signaling is of major importance in the cardiovascular system. There is close spatial proximity between surface mesenchymal PTHR1 and epithelial/endothelial sources of PTHrP in several organs, predominantly the kidney and bones [111,112]. Despite this paracrine coupling being expressed on a lower level in aortic and cardiac tissue, it has a tremendous impact on early embryonic development. Murine specimens systemically lacking expression of PTHR1 rapidly died during the embryonic phase due to cardiomyocyte failure [113]. The antiapoptotic potential of PTHR1 activation extends to osteocytes and hepatocytes, although the latter is likely explained by cardiomyocyte survival and the limitation of right-heart congestion [91,113]. The potential postnatal benefit of PTHR1 signaling was demonstrated by employing a canine model of myocardial infarction [114]. Administration of PTH (1–34) rescued left ventricular function, thereby delaying the installation of cardiogenic shock. Consistent with these findings, it has been proven that there are specific types of endothelial cells providing paracrine signaling under ischemic conditions, resulting in enhancement of both inotropy and lusitropy. PTHrP/PTHR1 signaling also improved coronary blood flow through the same epithelial-to-mesenchymal crosstalk [115]. 

PTH has long been recognized as a potent vasodilator under acute infusion settings across several vascular beds and various vertebrate species, including humans [116,117]. There is a widespread variation in the vasodilatory potential exhibited by PTH across multiple vertebrate vascular environments, presumably due to differences in local expression of co-adapter scaffolding proteins, NHERFs [118]. However, to the date of this writing, the exact mechanisms underlying temporal and spatial variances in PTH-mediated vasodilation are unknown. Endothelial production of NO is just partly responsible for the noticed increase in blood flow, but its presence is mandatory for the vascular smooth muscle cells (VSMC) to drive the rest of the response to PTHrP/PTH [119]. This resulting vasodilation is a possible mechanism for improved survival of cardiomyocytes in ischemic settings, aside from the antiapoptotic behavior expressed in paracrine fashion [115]. On a renal level, augmented expression of PTH/PTHrP in VSMCs leads to a sustained reduction in systemic blood pressure, along with improved filtration in response to volume loading [120]. Conversely, postnatal deletion of PTHrP in rodent VSMCs did not result in altered baseline blood pressure, but it disabled renal perfusion regulation in response to hemodynamic challenge [121].

### 8.2. Paradoxically, Hyperparathyroidism Does Not Manifest the Benefits of PTH Signaling

Despite the apparent beneficial effect exhibited by PTH signaling, the reality of both primary and secondary hyperparathyroidism [122,123,124] is vastly different. Primary hyperparathyroidism (PHPT) patients frequently exhibit impaired global left ventricular dysfunction, reduced coronary blood flow reserve and a shorter QT interval on electrocardiogram, accounting for an increased risk of ventricular arrhythmia [125]. Cardiovascular morbidity and mortality are important not only in PHPT but also in SHPT. A 2.2-fold higher risk of major adverse cardiac events was noted in incident SHPT (compared with healthy individuals), together with a 1.3-fold increase in risk of death over medium-term follow-up [124]. 

The unexpected behavior under conditions of prolonged PTH exposure was first proven through experimental studies and has been known for more than 35 years. Following maximal contraction of isolated femoral artery from healthy rodent subjects, successive administrations of PTH over tens of minutes were employed in order to evaluate the decline in vasodilation response. As early as the third administration, the relaxation potential attributed to PTH signaling was severely blunted, but overall arterial responsivity proved intact, as demonstrated by complete vasodilation following acetylcholine treatment [126]. The arterial tachyphylaxis also extends to renovascular territory, as proved by an isolated model of rabbity kidney [127]. 

The most probable explanation for the previously mentioned discordance is represented by rapid vascular desensitization in response to continuously high levels of PTH [127]. Aside from liver and kidney uptake, tissue endopeptidase proteolysis partly accounts for a time-dependent reduction in circulating levels of PTH, provided there is no continuous release source. The resulting degradation fragments, specifically PTH (7–84), may work against the canonical effects of PTHR1 activation. Aside from competitive binding to the active site of PTHR1, the biological action exerted by certain NH2-truncated PTH fragments plays a more significant role in the desensitization phenomena. Upon binding to PTHR1, albeit probably not the classical receptor site, the NH2-terminal degradation products promote receptor internalization and downregulation, employing neither PKA nor PKC pathways [128,129]. Furthermore, the existence of a novel C-terminal PTH receptor, with high affinity towards the COOH-terminal degradation fragment of PTH, has been assigned as part of the resistance mechanism occurring in uremic conditions [130].

### 8.3. The Cardiovascular “Snowball Effect” of Secondary Hyperparathyroidism

#### 8.3.1. Aldosterone/PTH Vicious Synergic Coupling

Early studies involving continuous PTH perfusion in humans returned deleterious systemic effects, with persistent hypertension and hypercalcemia being the most notable [131]. Apart from a rapid desensitization of the PTHR1 axis, several animal studies have proven both a direct and indirect effect of PTH on the secretion of Aldosterone (Ald), a key element in vascular tone modulation. Following stimulation by Angiotensin II (Ang II), there is an activation of the steroidogenic cascade in glomerulosa cells, primarily through an increase in both capacitive and voltage-gated calcium influx [132]. The response of bovine adrenal glomerulosa cells to Ang II when exposed to PTH was nearly double compared to the response in the absence of PTH [133]. PTH demonstrates a dose-dependent increase in intracellular calcium even in the absence of Ang II. An ionophore-like activity was attributed to the paracrine couple PTHR1/PTHrP, explaining the aforementioned effect on the Renin–Angiotensin II–Aldosterone (RAS) axis [134]. The pro-hypertensive effect was also noted in humans, whereupon binding to PTHR1, an increase in activities of both PKA and PKC pathways translates into the production of Ald and cortisol [135]. 

The relationship between Ald and PTH is more intricate than it transpires. Patients exhibiting persistently high levels of Ald, as is the case with Primary Hyperaldosteronism, suffer the consequences of increased calciuresis: hypocalcemic tendency, renal calculi and secondary hyperparathyroidism [136]. In addition, patients with Primary Hyperaldosteronism displayed higher levels of PTH and lower plasma calcium in comparison to essential hypertension (a pathology where the ratio Ald/Ang II is elevated, but not as much) [137]. Treatment targeted towards lowering Ald concentration should, therefore, bring PTH levels to normal. Spironolactone administration and surgical ablation of adenoma in Primary Hyperaldosteronism patients both brought PTH and calcium serum levels towards the normal range (while not necessarily inside the normal range) [137]. Despite the intricate PTH–Vitamin D–FGF23 axis, surgical removal of parathyroid gland adenoma maintained a statistically significant decrease in PTH concentration even after considering Vitamin D levels [138].

#### 8.3.2. High Levels of PTH Allow for Increased Renin Plasma Activity and Secretion of Angiotensin II: A Closed Loop

Adding to the dual-way synergic relation between PTH and Ald, renin responds in a drastically similar manner to chronically elevated PTH. Renin secretion is controlled by modulating the plasma concentration of calcium, the universal cation signal transductor. An acute increase in circulating calcium leads to activation of the calcium-sensing receptor in renal juxtaglomerular murine cells, followed by a drop in renin secretion, especially notable under low-salt/high-renin regimes [139]. However, chronic stimulation of the human calcium-sensing receptor—typical but not absolute occurrence under conditions of prolonged hyperparathyroidism—constantly yields higher renin plasma activity, albeit variable with salt intake [140]. In this regard, studies show a slight variability in the extent of renin activity increase without clearly stating whether there is solely an increase in secretion or a further secondary step augmenting plasma renin activity [141,142]. Infusion of Angiotensin II on human subjects—an intervention physiologically resembled by increasing plasma renin activity—results in a dose-dependent increase in plasma PTH level, quantifiable as soon as one hour following the injection [143]. The precise involvement of Ang II in determining PTH is strengthened by the dramatic reversal of effects after administering an Angiotensin-converting enzyme inhibitor, captopril, and lack of acute response following Ald infusion.

**Figure 3:**
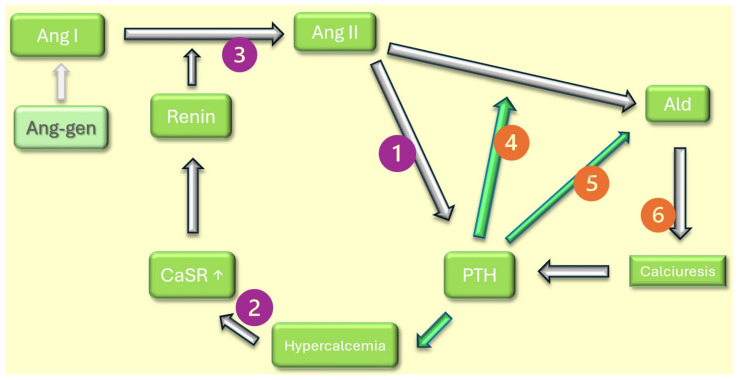
A schematic representation of the feed-forward cycles fueled by PTH. Ang-gen, Angiotensinogen; Ang II, Angiotensin II; CaSR, calcium-sensing receptor; Ald, Aldosterone. To the left of the figure, Ang II determines a dose-dependent response of Ald secretion (arrow no. 1). The progressively increasing PTH leads to a tendency to hypercalcemia, which increases the sensitivity of calcium-sensing receptor, translated into increased renin secretion (process 2). Angiotensin I conversion by renin closes the loop (arrow 3). To the right side of the figure, PTH acts both directly and indirectly on Aldosterone secretion by augmenting the signal of Ang II on glomerulosa cells (process 4 and 5, respectively). Conversely, Aldosterone determines calciuresis, the hypocalcemic tendency ultimately upregulating PTH and closing the loop (process 6).

Consistent with the bi-directional “feed-forward” cycles PTH is involved in, as resumed in Figure 3, a faster cardiovascular decline is expected in patients with SHPT. Therapy with inhibitors of the RAS axis reduces PTH levels in the human population, while the lack of this medication correlated with a more rapid progress in valvular calcification during the Japanese Aortic Stenosis Study [144,145].

The expression of PTHR1/PTHrP is essential both during the embryological development phase and postnatal, owing to the systemic antiapoptotic and vasodilating potential of the couple. The persistently high serum PTH and the accumulation of degradation fragments, a normal occurrence in CKD stages G2-G5, lead to desensitization of PTHR1, reducing the overall vasorelaxation potential on a systemic scale. PTH also influences vascular tone by interfering with the RAS axis. There is strong evidence for PTH as a central element in two vicious autocatalytic cycles, each one involving two consecutive elements of the aforementioned axis. Pharmacological intervention upon the RAS axis is advisable since both spironolactone and Angiotensin-converting enzyme inhibitors proved consistent reduction in PTH and calcium levels, thereby diminishing the toxic potential of elevated PTH and delaying surgical treatment of valvular calcification.

### 8.4. Vascular Calcification—The Implication of PTH

Despite being moderately prevalent in 2% of the population over the age of 60, aortic valve calcification bears tremendous cardiovascular mortality [146]. The precise role of PTHR1/PTHrP in human cardiac valve disease is not fully understood at the moment. However, the paracrine couple shows important embryological significance since knockdown in zebrafish significantly altered aortic valve morphogenesis [147]. An in vitro calcification model demonstrated the role of PTH in promoting osteoblastic differentiation of human endothelial cells, as proven by an increase in bone morphogenetic proteins 2 and 4 [148]. This effect was attained through direct signaling via the extracellular signal-regulated protein kinase 1/2 and nuclear factor-κB signaling pathways. 

Cardiac valve calcification has been extensively studied in patients with PHPT, less so in conditions like CKD—prototypical SHPT. Nonetheless, a rapid progression of the mineralization process has been attributed to a series of independent risk factors: elevated PTH, hypertension and osteoporosis [149,150]. Alongside the persistent rise in PTH levels as soon as stage G2, CKD harbors a whole array of other cardiovascular risk factors, possibly adding to the profile of an SHPT patient [10,151]. Calcimimetics is a calcium analog medication that efficiently decreases PTH secretion by activating the calcium-sensing receptor. The “pharmacological parathyroidectomy” action of this class of medication leads to improved prognosis, augmenting the role played by PTH in vascular and aortic valve calcification [152,153,154]. 

Controlling the “mineral transfer” is a must in order to delay surgical intervention, a requirement in advanced valvular disease [155]. Surgical valve treatment in asymptomatic HPT is, until present, not standard. If it would eventually prove beneficial, setting an age-specific cutoff may be required in hyperparathyroidism—both primary and secondary—patients developing valvular calcification [156].

### 8.5. The Key to Assessing Vascular PTH Axis Status Could Lie in the Endothelial Involvement in PTH Signaling

Intact endothelial NO production may be, by far, the most significant contributor to vessel dilation capacity, especially in bone vasculature, since singular blockade of NO synthase leads to a dramatic 80% reduction in vasodilation response, while denudation completely abolishes it in murine arteries [119]. Augmented VEGF strengthens the vasodilating response, as demonstrated by a drop of 38% after administration of an anti-VEGF antibody [157]. VEGF also enables closer proximity of bone marrow vasculature to the new bone-forming surfaces [158]. The fact that mRNA expression of VEGF is dependent not only on both PKA and PKC activities but also on several NO-dependent pathways suggests a highly intricate network of PTHR1 downstream signaling, which certainly extends to the human species [159]. 

Histomorphometric assessment of osteoblast and mineralizing surfaces, together with the level of circulating intact PTH, could indicate the momentarily skeletal tissue responsiveness towards PTH (1–84). The local changes in blood flow closely follow the osseous anabolic effects in response to PTH in mice specimens [160]. Under the assumption of relatively uniform—regarding endothelial status—arterial beds throughout the body, skeletal anabolic status may serve as a reliable substitute for vascular health [161]. Foreseeably, systemic arteriosclerotic dysfunction (essentially defined by endothelial dysfunction) reduces the signaling potential of PTHR1/iPTH in skeletal vasculature, translating into reduced hip bone mass in humans [162]. The direct correlation between vascular and skeletal health holds in human subjects undergoing dialysis. Despite the uremic milieu and highly variable amplitude of endothelial toxicity, the ankle-brachial index indicator of peripheral arterial disease correlated to surface mineralization [161]. Despite the extensive involvement of endothelium in enabling full PTHR1 activation, the skeletal anabolic state is not yet a reliable marker for cardiovascular PTHR1 status.

## 9. Management of Biochemical Abnormalities in CKD-MBD Focusing on PTH Level Changes

### 9.1. Limitation of Phosphate Retention Attenuates PTH Levels

Serum phosphate, the most important trigger in MBD development and progression, should ideally be maintained inside the normal range [19]. Possible strategies include limiting diet intake, use of binding agents, or improved elimination in hemodialyzed patients. Western diets are especially rich in Pi due to the higher proportion of meat and milk, as well as the additives used in processing [163]. Several binding agents are available, but the non-calcium-based ones, such as sevelamer, display a better outcome on bone turnover than calcium salts [164]. There is an independent association between binder treatment and decreased serum Pi, circulating intact PTH, and reduced risk of mortality [165,166]. Excessive administration of calcium-based binders is likely as dangerous in any stage of CKD since an increase in calcium balance poses a risk for vascular calcification [167]. 

Despite their demonstrated benefits, phosphate binders should be administered only to carefully selected patients and in such a manner that avoids hypercalcemia to the greatest extent possible. 

### 9.2. Manipulation of Calcium-Sensing Receptor Yields Beneficial Effects on Progression of Mineral and Bone Disorder

Activation of CaSR through either increased circulating calcium or calcimimetics decreases the expression of the PTH gene in models of SHPT and uremic conditions [168]. Care should be taken when administering calcium since a daily oral dose of 1000 mg/day is enough to achieve a neutral calcium balance. Supplementation in patients with adequate calcium in their diet is not recommended [167]. The other way of activating CaSR is using cinacalcet. Before attempting to reduce PTH, calcitriol levels should first be normalized through supplementation since Vitamin D deficiency could induce SHPT solely [169]. However, if PTH levels remain high after normalization of Vitamin D activity (cut-off point for insufficiency at 30 nmol/L), calcimimetics may represent an alternative [19]. Cinacalcet administration in stage 5 dialyzed CKD subjects is recognized to increase bone mineral density through a significant reduction in PTH levels. Despite the strong effect of the treatment towards beneficial changes in MBD (reduced risk of fracture and cardiovascular events), there was no reported association between PTH levels and BMD [152,170]. Likely, the lack of direct correlation between PTH levels and MBD improvement lies in the changes exhibited by another fundamental MBD marker during the course of treatment, FGF-23 [171]. 

Administration of calcimimetics should be carefully judged since strong inhibition of PTH release can lead to more frequent hypocalcemic episodes, especially when employing more efficient CaSR agonists [152]. The effect of calcimimetics on overstimulated and hypertrophied parathyroid cells is largely limited. Kidney-transplanted patients with tertiary HPT displayed better efficiency in reducing PTH levels by surgical approach rather than treatment with cinacalcet [172]. 

Manipulation of calcium is also useful in dialyzed patients. In a small proportion of these subjects, constantly elevated calcium levels may be encountered. Adynamic bone disease, a variant of renal osteodystrophy, is the result of persistent inhibition of PTH release, which in turn is determined by constantly increased serum calcium levels. Using low-calcium dialysate solutions, a lower frequency of hypercalcemia episodes is expected, followed by release of the inhibition in PTH expression and return to the expected—higher than normal range—plasmatic concentrations. The increase in PTH restores the osteogenic potential and draws a parallel increase in bone turnover [173].

Once Vitamin D levels have been normalized, the calcium-sensing receptor may represent a target in order to reduce the circulating levels of PTH. Despite the temptation of administering high doses of Calcium or calcimimetics, practitioners have to acknowledge the risk of adynamic bone disease brought by conspicuously low PTH.

### 9.3. Vitamin D Supplementation Yields Disparate Results Depending on the Analogue

Both hydroxylated metabolites of cholecalciferol—calcifediol and calcitriol—act on bone metabolism. CKD patients constantly exhibit insufficiency or even deficiency in cholecalciferol levels, therefore pushing practitioners to recommend supplementation with D3. The recommended daily dose of 800 IU D3 plays a role in delaying or ameliorating SHPT across all-stages CKD patients, reduces PTH levels in dialyzed individuals and decreases inflammatory markers [174,175]. However, administration of products located further down the synthesis cascade of calcitriol yields various results. Calcitriol or its analog paricalcitol, the more biologically active form of Vitamin D, decreases PTH and bone alkaline phosphatase but yields no change in bone mineralization when administered in G3-5 CKD or dialyzed patients [176,177]. The opposite is true in calcidiol supplementation, the metabolite previous to calcitriol, which improves mineralization but does not influence PTH levels. 

The different outcomes of the hydroxylated D3 metabolites point towards likely different signaling pathways activated by molecules belonging to the same class, Vitamin D. Baseline levels of Vitamin D inversely correlate with the risk of all-cause and cardiovascular mortality in pre-dialysis CKD patients [178]. Despite slightly different outcomes of administering different hydroxylated D3 products, recent studies discourage indiscriminate supplementation of Vitamin D, both in the general population and CKD patients. There is no certainty regarding the benefits of Vitamin D on any organ, apart from reduced fracture risk [38,179]. 

### 9.4. Anabolic Agents

Intermittent infusion of PTH enhances bone mineralization through classical signaling on PTHR1. The efficacy of synthetic analogues like Teriparatide and Abaloparatide in ameliorating MBD is not yet definitive since many of the studies excluded patients with abnormal PTH levels. However, in these cases, daily or weekly administration of Teriparatide improved bone mineral density in each of the renal function groups, including hemodialysis [180,181]. The safety of this drug is highlighted indiscriminately with respect to PTH administration to patients in stages G4 and G5 CKD, who presumably displayed higher than normal PTH concentrations [182]. Despite the limited number of studies and the strict inclusion criteria, Teriparatide was the pharmaceutical option that displayed the highest efficacy (sharing the spot with Denosumab) in improving BMD in classical sites, vertebras and femoral necks [183]. Larger studies are needed in order to demonstrate efficacy in CKD-MBD regardless of PTH levels [184].

### 9.5. The Lower the PTH Levels, the Better? Not in Every Circumstance

Both Vitamin D activity and PTH levels play a massive role in the pathogenesis of end-stage CKD; therefore, treatment should aggressively target the two signaling cascades. However, hemodialysis patients with a lower average intact serum PTH display a significantly higher risk of non-fatal cardiovascular events compared to patients with serum PTH values in the range of secondary hyperparathyroidism [185]. The cut-off points chosen were 60 pg/mL for low PTH and 600 pg/mL for the low limit of values in the SHPT range. Achieving PTH values in the low-PTH range following parathyroidectomy did not yield the same clinical outcome. Consistent with these findings is the recommendation of using only low-dose Vitamin D receptor agonist, Paricalcitol [186].

The poor outcome in the low-PTH subjects likely arises from the low-turnover bone state. In this condition, mineral deposition is mostly favored in extra-skeletal tissues, leading to the worsening of vascular calcification and consequent cardiovascular failure [187].

## 10. NHERF1—In the Shadow of PTH Signaling

### 10.1. The Physiological Coupling of Scaffolding Protein NHERF1 and PTHR1

PTHR1 belongs to the family of G-protein coupled receptors (GPCRs), the largest family of receptors in vertebrates. The vast majority of GPCRs convey the signal through a downstream modulation of either adenylyl cyclase (AC) or phospholipase C (PLC). PTHR1 defies the aforementioned statement, employing both AC and PLC, sometimes at the same time. As such, in vascular smooth muscle cells (VSMCs), PTH stimulates only AC; in cardiac myocytes, PTH activates PLC, while in osteoblasts, both complexes are upregulated following signaling [188,189,190]. The structure of PTHR1 ends in a domain able to recognize a specific molecular pattern, known as the ETVM motif, designed to recognize the PDZ group of proteins. PDZ group of proteins was named after the structural similitude between the postsynaptic density protein (PSD95), Drosophila disc large tumor suppressor (DlgA) and zonula occludens-1 protein (ZO-1). The PDZ proteins pertaining to PTHR1 are sodium–hydrogen exchanger regulatory factor-1 and 2, NHERF1 and NHERF2, respectively [191]. The signaling pathways chosen following PTH coupling are determined by the scaffolding protein subtype present at the intracellular binding site. As such, PTHR1 naturally employs AC cascade, switching to PLC only in the presence of NHERF2 [192]. NHERF1 is constitutively expressed along PTHR1, ensuring adequate downstream signaling following receptor internalization, enhanced ligand selectivity and delayed desensitization.

Adequate PTHR1 signaling depends on the subtype of scaffolding protein expressed on the intracellular domain of the receptor. The importance of NHERF scaffolding proteins is not limited only to cascade switching. NHERF1 acts as a molecular filter at the PTHR1 active site by allowing only a few of the PTH hormone short-length variants to bind and internalize the receptor, thereby fully activating downstream signaling. Physiologically, cathepsin proteases in parathyroid glands truncate PTH on its amino end, resulting in the PTH (7–84) variant, which, in situations of hyperparathyroidism (such as advanced CKD), could accumulate to a dangerous extent. NHERF1 prevents binding of this by-product to PTHR1 [128]. Moreover, the presence of NHERF1 ensures the inhibition of receptor desensitization by interfering with β-arrestin [193]. In order for PTH to correctly transduce the signal to AC and further downstream, the intact structure of NHERF1 has to be expressed. An altered scaffold protein variant in human kidney cells would lead to the amplification of the normal AC activity resulting from PTHR1 internalization, accounting for several phenotypes of mineral ion disorders [194]. All things considered, NHERF1 is a fundamental component in conveying PTH signals.

### 10.2. Reduced Expression of NHERF1 Leads the Way for Various Renal Alterations

Dopamine regulation of ion balance is of significant importance at the renal level. Activation of dopamine receptor subtype 4 should directly inhibit Na/K ATPase (NKA) in the brush border of the proximal renal tubule [195]. Loss of NHERF1 in the spontaneous hypertensive rat model is incriminated in the impaired dopamine regulation of NKA characterizing the phenotype [196]. While NHERF1 transfection restored dopamine signaling, this could represent a mechanism for the progression of hypertensive disease in NHERF1-deficient subjects. An even more valuable model for NHERF1 deficiency is provided by aging, particularly the F344 rodent model [196]. Since CKD is fundamentally described by accelerated aging, there is potentially a decrease in total renal NHERF1 in human subjects. The F344 aging rodent model displayed increased renal fibrosis, determined by reduced inhibition of transforming growth factor-β1-mediated epithelial to mesenchymal transition [197]. In addition, loss of NHERF1 is also involved in reduced resistance to nephrotoxins. Following acute exposure to cisplatin, NHERF1 knockout rodents displayed a higher decline in renal function and histological injury scores compared to wild-type animals, underlining the importance of NHERF1 in acute inflammatory settings [198]. The susceptibility to damage is backed up by findings of increased inflammatory markers (TNFα) in renal tissue of NHERF1-knockdown old rodents, which have not been specifically exposed to nephrotoxins [199]. Lastly, the involvement of NHERF1 as a responder in chronic inflammation is unveiled by an increase in apical expression of the molecule—albeit no change in total expression in renal lysate—in diet-induced CKD in rodents [200].

To the date of writing this review, to our knowledge, there is no article analyzing the profile of NHERFs in the course of chronic kidney disease in humans, nor its role in the progression of renal function decline. However, the implication of NHERF1 in aging, inflammation, fibrosis and injury response may provide insight into the development of CKD. NHERF1 is a central player in the inflammatory response arising from chronic insults, such as aging and hypertension [201]. There are still many missing pieces of the puzzle, for instance, the profile of molecule expression according to age, assessment of whether NHERF1 is a responder or the mediator in renal injury, and if NHERF1 overall tissular levels correlate with disease severity.

## 11. Discussions

Chronic kidney disease is one of the most prevalent non-communicable diseases [1]. As early as stage G2 in the progression of the disease, there is a tendency towards phosphate retention, which is counteracted both by increasing phosphaturia and reducing intestinal and skeletal reabsorption [10]. As part of the parathormone–Vitamin D–Fibroblast Growth Factor-23 axis, changes in PTH level act as a compensatory mechanism in nearly all phases of CKD in an attempt to rescue the normal plasma phosphate level. While FGF-23 may increase in concentration earlier than PTH on the scale of CKD progression, measurement of PTH is more reliable, cheaper, faster and available on a wider scale [42,46]. Therefore, PTH is deemed a powerful marker for the complication rate starting from stage G3 CKD, specifically when interpreted as the plasmatic trend in association with glomerular filtration rate and clinical presentation [19,51,110].

CKD and the associated uremic milieu, particularly in the latter stages of the disease, takes its toll on the intestinal microbiota [64]. While PTH displays a bimodal behavior regarding bone homeostasis, alteration of the microbiota towards the preponderance of short-stranded filamentous bacteria enables local expansion of T-helper cells subtype 17 [52]. Exogenous supplementation with butyrate, a normally occurring metabolite in a healthy individual’s gut, manages to rescue the function of regulatory T cells and restore the osteoformatory action of PTH [52]. There is limited evidence for gut flora restoration using Lactobacillus species [70].

Regarding bone metabolism, the persistent increase in PTH levels drags a rise in both Dkk1 and sclerostin concentrations [82,86]. The impact of the latter hormones may often be negligible compared to the effect of PTH [82]. The clinical outcome of persistently elevated PTH is any of the following: osteitis fibrosa cystica, osteomalacia and adynamic bone disease [94]. Conversely, excess lowering of the PTH level by indiscriminate use of calcimimetics and Vitamin D could put the patient at a high risk of fracture, particularly when developing adynamic bone disease [95]. Furthermore, lower-than-expected serum PTH in dialysis patients also returned an increased risk for non-fatal cardiovascular events [185].

Despite the fundamental role played by PTH during the embryological phase, the consistently elevated concentrations of PTH in CKD result in effects opposing those expected. The most likely explanation is represented by a rapidly installed tachyphylaxis [126]. Apart from a reduced vasodilatory potential of any vascular bed, vascular and valvular calcification are other elements that significantly stress the cardiomyocytes in uremic conditions [148]. On top of that, the RAS axis engages in a vicious self-catalyzed cycle of upregulation [121,135]. The signaling potential of the paracrine couple PTHR1/PTHrP could serve as the link between bone formation capacity and cardiovascular health status [161]. 

Last but not least, the scaffolding protein NHERF1 could prove a potential future target for the reduction of renal fibrosis and inflammation, increasing the resistance to endogenous and exogenous nephrotoxins and even controlling hypertension [198]. It is known that a decline in NHERF1 expression is a marker of aging [201]. However, there is inconclusive human evidence regarding whether this molecule is either a sole marker of the disease or a consequence of the damage.

## 12. Conclusions and Future Directions

The present review is aimed at revisiting the mechanisms employed by parathormone in determining the characteristic multiple organ damage in the context of chronic kidney disease. Being a key component of the parathormone–Vitamin D–FGF-23 axis, PTH displays increased plasmatic concentrations as soon as stage G2 CKD as part of mineral and bone disorder. Aside from the classical involvement in skeletal homeostasis, PTH manifests deleterious effects indirectly on the microbiota and cardiac pump function and directly on vascular beds on a systemic scale. PTH proves to be a reliable and widely accessible marker for complication rate while being less accurate for disease progression. Interventions toward reducing serum PTH should be approached with limited enthusiasm since a lower-than-expected plasma PTH concentration could put patients at risk for cardiovascular events.

Attributing a certain observed effect to the change in a singular parameter is challenging, considering the multitude of both adaptive and destructive changes undergone during the progression of CKD. Altering PTH concentration most likely determines a change in the FGF-23–Vitamin D–PTH axis, limiting the association of effect to the hormone. Another limitation is represented by the impossibility of translating findings from primary HPT patients to CKD subjects due to the uremic milieu the latter are constantly exposed to. In light of PTH determining several deleterious systemic effects, it is advisable for practitioners to frequently monitor the level and trend of this hormone in their CKD patients, starting as early as G2, along with serum calcium and phosphate. Adynamic bone disease should definitely be avoided. Diet and pro-biotic treatment may be advisable, especially in patients whose digestive symptoms are tolerated. Surgical treatment of valvular disease could be advised from an earlier age when a patient is more likely to undergo the procedure successfully. Lastly, pharmacological manipulation of mineral metabolism should be carefully judged since indiscriminate administration of Vitamin D or phosphate binders could throw off mineral balance, posing a high risk for an adynamic bone state.
